# First identification of human adenovirus subtype 21a in Shenzhen, China with high-throughput sequencing

**DOI:** 10.3389/fmicb.2025.1692162

**Published:** 2025-10-13

**Authors:** Dan-dan Niu, Shi-song Fang, Zhi-gao Chen, Qiu-ying Lv, Ting-ting Liu, Ni-xuan Chen, Ying-ying Li, Ying Sun, Chao Li, Shun-wu Huang, Yan-peng Cheng, Hong-lin Wang, Ying Wen, Yi-xiong Chen, Xin-dong Zhang, Jian-hua Lu, Xiao-lu Shi, Zhen Zhang, Xuan Zou, Tie-jian Feng

**Affiliations:** ^1^Shenzhen Center for Disease Control and Prevention, Shenzhen, Guangdong Province, China; ^2^Baoan Center for Disease Control and Prevention, Shenzhen, Guangdong Province, China; ^3^Shenzhen Research Center for Communicable Disease Control and Prevention, Chinese Academy of Medical Sciences, Shenzhen, Guangdong Province, China

**Keywords:** adenovirus type 21, whole genome, phylogenetic analysis, variation, Shenzhen

## Abstract

Human adenovirus type 21 (HAdV-21) is recognized as an important pathogen responsible for acute respiratory infections (ARIs). However, it has been rarely reported and remains poorly characterized to date in China. Outpatient or inpatient children under 14 years old with suspected ARIs were enrolled from two hospitals in Shenzhen from September 2023 to April 2024. Respiratory samples were collected and tested for 22 common respiratory pathogens. A comparative analysis was conducted on the positive proportions of pathogens among different groups. Phylogenetic analysis and amino acid mutation analysis were conducted for HAdV-21 strains. A total of 498 pediatric patients with ARIs were enrolled. There were 366 (73.5%) patients infected with at least one pathogen, and 133 (26.7%) patients co-infected with other pathogens. The most frequently detected pathogens were *streptococcus pneumoniae* (*S. pneumoniae*) (30.7%, 153/498), HAdV (16.7%, 83/498), and influenza virus (IFV) (16.5%, 82/498). The positive HAdV-21 strain was sequenced and classified as subtype 21a with genome closely related to other strains found in China, and compared with HAdV-21 strains GZ09107, GZ06109 and BB/201903 in China, Shenzhen-2024-5-ILI-1109 contained only one amino acid insertion mutation in the penton base (GTT, Valine). Phylogenetic analysis for whole genome and major antigen proteins showed that global HAdV-21 strains could be classified into two branches, branch 1 including genotype 21p, branch 2 including subtype 21a and 21b. There were three highly variable regions (HVR3, HVR4, and HVR7) in the hexon protein that varied between two branches. This study initially reported a case of HAdV-21a infection in children in Shenzhen, and the genome showed one amino acid insertion mutation in the penton base compared with reported HAdV-21 strains in China. Our findings may contribute to a better understanding of the molecular epidemiological characteristics of HAdV-21 strains, as well as aid in the development of vaccines.

## Introduction

Human adenovirus (HAdV) is a non-enveloped, double-stranded DNA virus classified within the genus Mastadenovirus of the family Adenoviridae (Nemerow et al., 2012). According to the different tissue preferences, HAdV is divided into 7 species (A-G) and associated with a broad spectrum of clinical diseases, such as acute respiratory infections (ARIs), gastrointestinal infections, conjunctivitis, and obesity (da Silva Fernandes et al., 2021; Lynch et al., 2011). Species B (such as HAdV-3, 7, 55), C (such as HAdV-1, 2, 5), and E (HAdV-4) are most closely related to ARIs in children (Mao et al., 2022; Wo et al., 2015).

HAdV type 21 is classified under species B and was initially identified in 1956 (Bell et al., 1959). The prototype strain was isolated from a one-year-old child presenting with both trachoma and conjunctivitis in Saudi Arabia. HAdV-21 was later found to be associated with a variety of diseases, including ARIs (James et al., 1979), severe pneumonia (Philo et al., 2018), myocarditis (Kühl et al., 2005), conjunctivitis (Darougar et al., 1978), hemorrhagic cystitis (Oltolini et al., 2020), encephalitis (Mukai and Bowers, 1975), and even fatal infections in both pediatric and adult patients (Hage et al., 2014). There was an increased risk (odds ratio, 7.6; 95% confidence interval, 2.6–22.3) of severe disease compared with the more common HAdV-3 infections (Gray et al., 2007), and an increasing trend of HAdV-21 detection over time. The circulation of ARIs associated with HAdV-21 has been documented among military recruits and civilian populations in several developed countries (Kajon et al., 2015; Pearson et al., 1981), and has led to nosocomial infections outbreaks in lung transplant recipients at a large tertiary care hospital (Philo et al., 2018). However, data regarding HAdV-21 remain limited, and only a few cases of HAdV-21 infection have been reported in North America, Europe, and Asia. In addition, reports of HAdV-21 infections were particularly scarce in China. In March 2019, the first case of HAdV-21a in China was identified in a 20-year-old male outpatient presenting with fever and sore throat, using both MinION and Illumina sequencing platforms (Ye et al., 2020). Subsequently, two additional cases of HAdV-21 infection were diagnosed in Guangzhou, China, in 2019, both presenting with severe lower respiratory tract illness (Liu et al., 2022).

A comprehensive understanding of HAdV-21 epidemiology relies on regional infection data. We investigated the prevalence of HAdV among pediatric patients with ARIs in Shenzhen, China, during 2023–2024, and further characterized the clinical manifestations, phylogenetic relationships, amino acid variations, and antigenic profiles of the HAdV-21 strains among children.

## Materials and methods

### Participants

Outpatient or inpatient children under 14 years old with suspected ARIs from September 2023 to April 2024 were enrolled from two Grade-A Tertiary hospitals in Shenzhen. The departments mainly included emergency department, fever clinic, respiratory department, pediatrics department, intensive care unit (ICU), etc. The definition of patients with ARIs was according to the 2016 clinical practice guidelines by the Chinese Thoracic Society (Cao et al., 2018). Those with non-infectious diseases (asthma or cancer, etc.), without signed informed consent, and missing important data were excluded.

### Respiratory sample collection

Respiratory samples of children were collected before treatment from patients within 72 h of presentation, including nasopharyngeal swabs, sputum, bronchial or alveolar lavage fluid (Metlay et al., 2019). Children with upper respiratory tract infection were given priority to collect nasopharyngeal swabs, and with lower respiratory tract infection including pneumonia were given priority to collect bronchial or alveolar lavage fluid. About 30 samples were collected per week and meanwhile ensure the distribution balance and representativeness of samples. The samples were refrigerated at 2–8 °C in viral transport medium, transported on ice to the Laboratory of Shenzhen Center for Disease Control and Prevention, and analyzed immediately or stored at −80 °C before analysis. Meanwhile the sample submission forms were filled out, including information of sample collection time, sample type, etc.

### Screening for common respiratory pathogens

Viral nucleic acid extraction kit (qEx-DNA/RNA virus) and automatic nucleic acid extractor were used to extract nucleic acid, and then respiratory pathogens detection kit (GK-OP-030F-50T) was used to detect 22 pathogens and their subtypes using TaqMan real-time quantitative polymerase chain reaction (qPCR), including influenza virus (H1N1 and H3N2 subtype of IFV-A, the Victoria lineage and Yamagata lineage of IFV-B), SARS-CoV-2, human rhinovirus (HRV), HAdV, human respiratory syncytial virus (HRSV), human parainfluenza virus subtype 1–4 (HPIV), human metapneumovirus (HMPV), human bocavirus (HBoV), human coronavirus (OC43, 229E, HKU1, and NL63), *mycoplasma pneumoniae (M.pneumoniae)*, *streptococcus pneumoniae* (*S.pneumoniae*), enterovirus (EVs), and *chlamydia pneumoniae (C.pneumoniae)*. Laboratory testing was carried out in accordance with the Regulations on Biological Safety Management and the Standard Operating Procedure of National Pathogenic Microbial Laboratories.

### Type identification of HAdV based on three target genes

The published universal primers and methods were utilized to amplify three target gene sequences of the HAdV positive samples including Penton base (1,253 bp), Hexon (1,685 bp), and Fiber (1,153 bp) genes (Wu et al., 2022) with TaKaRa PCR amplification kit (R011), and the universal primers were shown in Supplementary Table 1. This technique covered 22 common serotypes of HAdV, including HAdV-A18, -B3, -B7, -B11, -B14, -B16, -B21, -B34, -B35, -B50, -B55, -B66, -B68, -C1, -C2, -C5, -C6, -D19, -D37, −E4, -F41, and -G52. The PCR products of HAdV positive samples were sent to Sangon Biotech (Shanghai) Co., Ltd. for three target gene sequencing (Adrian et al., 1986), which were sequenced in both directions to ensure the correctness of the sequence. Phylogenetic analysis was conducted based on the three target genes of HAdV to identify the type (Singh et al., 2012).

### Whole genome sequencing and annotation of HAdV-21 strain

The whole genome sequence of HAdV-21 infected strain was obtained using next-generation sequencing with Illumina sequencer. The raw sequencing data was evaluated for quality through FastQC, and the Illumina sequencing data was subjected to quality clipping through Trimmomatic to obtain relatively accurate and valid data. Next-generation sequencing data were spliced without or with parameters using SPAdes. Use GapFiller to fill the GAP of the contig obtained by splicing. Sequence correction was carried out using PrInSeS-G to correct base errors and the missing insertions of small fragments during the splicing process. Genetic elements were predicted using Prokka. RepeatMasker was used to identify repetitive sequences on the genome. The complete genome of the HAdV-21 strain was annotated based on HAdV-21 strain GZ09107 (accession number MW151243). Complete genome sequence of the HAdV-21 strain in this study was logged in the GenBank database with accession number PX101489.

### *In silico* restriction enzyme analysis

The REA of the HAdV-21 strains was performed with CisSERS (Sharpe et al., 2016). Multiple genomes included the current HAdV-21 genome, HAdV-21a NHRC-5, HAdV-21b OHT-006, and HAdV-21p AV-1645. Five restriction enzymes including BamHI, BglI, BglII, KpnI, and SmaI were chosen to recognize the restriction sites of each genome with default parameters. Gel images containing restriction sites and molecular weights were generated by CisSERS. Genome type designations were based on unique arrays of restriction profiles with a panel of 6 endonucleases.

### Phylogenetic analysis of HAdV

Commercial Sequencher 5.4.6 software (Genecode, United States) was used to splice and collate the original nucleotide sequences of whole genome, Penton base, Hexon and Fiber genes. The whole genome and three genes were intercepted and saved. The homologous nucleotide sequences were aligned using the Basic Local Alignment Search Tool (BLAST) program in the GenBank database established by the National Center for Biotechnology Information (NCBI) of the United States. Download the complete sequences of the whole genome and three genes of global HAdV strains from NCBI, and add the sequences of the virus strains studied in this study to construct phylogenetic trees. Using MAFFT 7.526 software^1^ for more sequence alignment. MEGA 12.0 software was used to analyze the genetic phylogenetic relationship and p-distance (Kumar et al., 2024). Neighbor-joining method (NJ) (Kimura2-parameter nt substitution model) and Maximum likelihood method (ML) (Tamura-Nei nt substitution model) were used, respectively. The bootstrap value was set to 1,000 to evaluate the reliability of the constructed phylogenetic trees (Tamura et al., 2021). Bioedit 7.0.4.1 software^2^ was used to calculate the differences in nucleotide and amino acid sequences of the whole genome and three genes of HAdV strains. The sequences of the whole genome, Penton base, Hexon, and Fiber genes of HAdV for phylogenetic analyses retrieved from GenBank and in this study were summarized in Supplementary Table 2.

### Statistical analysis

Descriptive analysis was conducted using frequency and percentage for qualitative variables, and mean±standard deviation (normal distribution), or median and interquartile range (skewed distribution) were used for quantitative variables. We classified children into two groups for analysis including children <5 years old and school-age children (5–14 years old). The positive proportions of pathogens among different groups were compared through Pearson *χ*^2^ test and bonferroni method. Statistical analysis was implemented in R 4.1.2. A two-sided *p* value of <0.05 was considered to indicate statistical significance.

## Results

### Basic information and detection proportions of multiple pathogens among participants

A total of 498 children with ARIs were enrolled from hospitals in Shenzhen from September 2023 to April 2024. The median and interquartile range of age was 5.0 (2.8, 7.0) years old, and 46.8% (233/498) were under 5 years old, 57.6% (287/498) were male, and 44.4% (221/498) were hospitalized.

**Figure 1:**
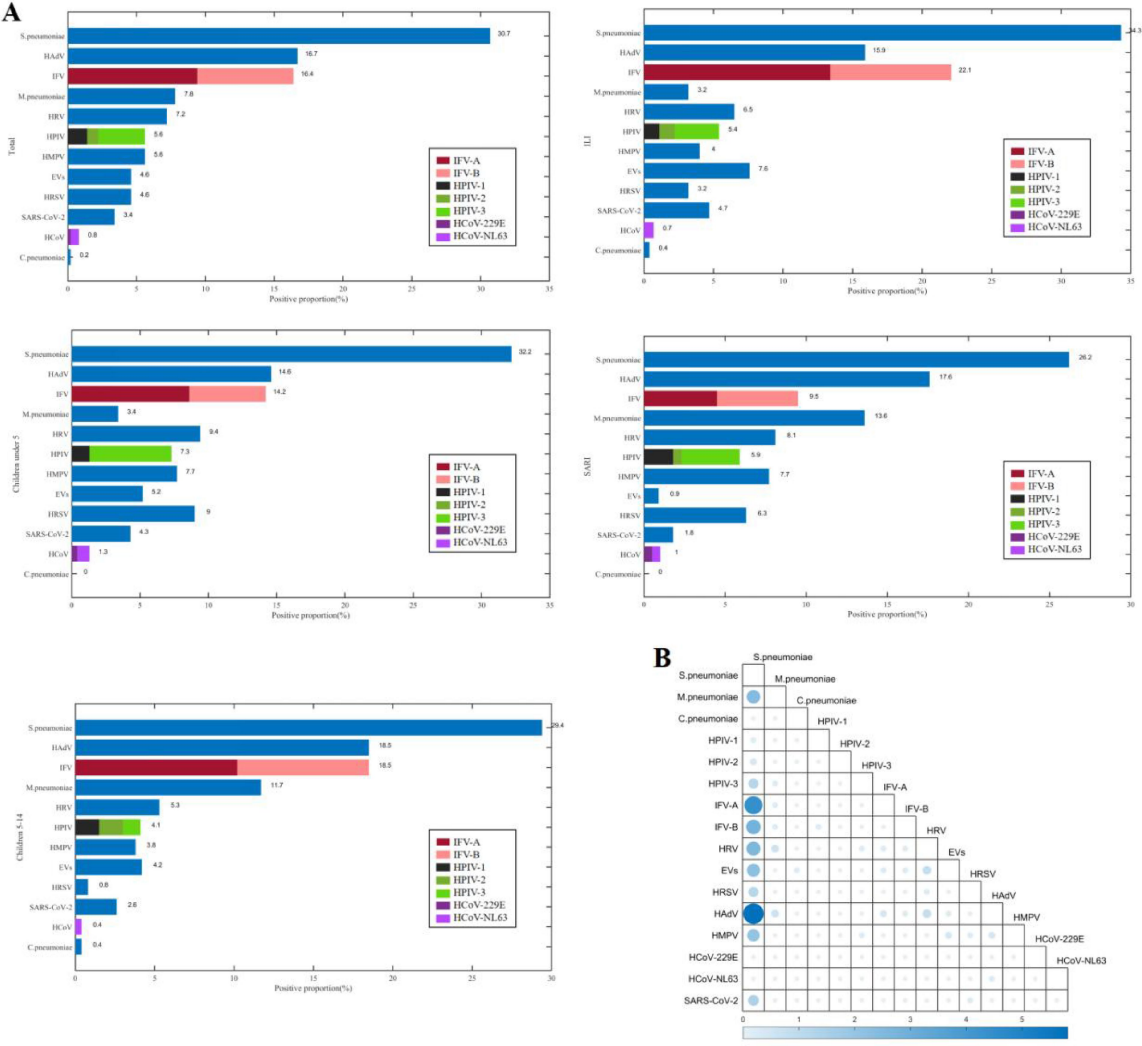
The positive proportions **(A)** and co-detection proportions **(B)** of respiratory pathogens among children from September 2023 to April 2024 in Shenzhen.

There were 366 (73.5%) patients infected with at least one pathogen, and 133 (26.7%) patients co-infected with other pathogens. The most frequently detected pathogens were *S. pneumoniae* (30.7%, 153/498), HAdV (16.7%, 83/498), and IFV (16.5%, 82/498) (Figure 1). The predominant pathogens in children under 5 years and those aged 5–14 years were *S. pneumoniae*, HAdV, and IFV. The top three pathogens were *S. pneumoniae*, IFV, and HAdV in the ILI (influenza-like illness) group, and *S. pneumoniae*, HAdV, and *M. pneumoniae* in the SARI (severe acute respiratory infections) group. The three most frequent co-infection combinations were *S. pneumoniae* with HAdV (5.8%, 29/498), with IFV-A (4.4%, 22/498), and with IFV-B (2.8%, 13/498).

The positive proportion of *M.pneumoniae* among children aged 5–14 years (11.7%, 31/265) was higher than that among children under 5 years (3.4%, 8/233) (*χ*^2^ = 11.733, *p* = 0.001), and the positive proportion of HPIV-3 among children under 5 years (6.0%, 14/233) was higher than that of children aged 5–14 years (1.1%, 3/265) (*χ*^2^ = 8.942, *p* = 0.003), the positive proportion of HRSV among children under 5 years (9.0%, 21/233) was higher than that among children aged 5–14 years (0.8%, 2/265) (*χ*^2^ = 19.194, *p* < 0.001). The positive proportion of HRSV among male children (6.6%, 19/287) was higher than that among female children (1.9%, 4/211) (*χ*^2^ = 6.161, *p* = 0.013). The positive proportion of *M.pneumoniae* among children with SARI (13.6%, 30/221) was higher than that among children with ILI (3.2%, 9/277) (*χ*^2^ = 18.157, *p* < 0.001), the positive proportion of IFV-A among children with ILI (13.4%, 37/277) was higher than that among children with SARI (4.5%, 10/221) (*χ*^2^ = 11.220, *p* = 0.001), the positive proportion of EVs among children with ILI (7.6%, 21/277) was higher than that among children with SARI (0.9%, 2/221) (*χ*^2^ = 12.438, *p <* 0.001).

### Detection characteristics and type identification of HAdV

**Figure 2:**
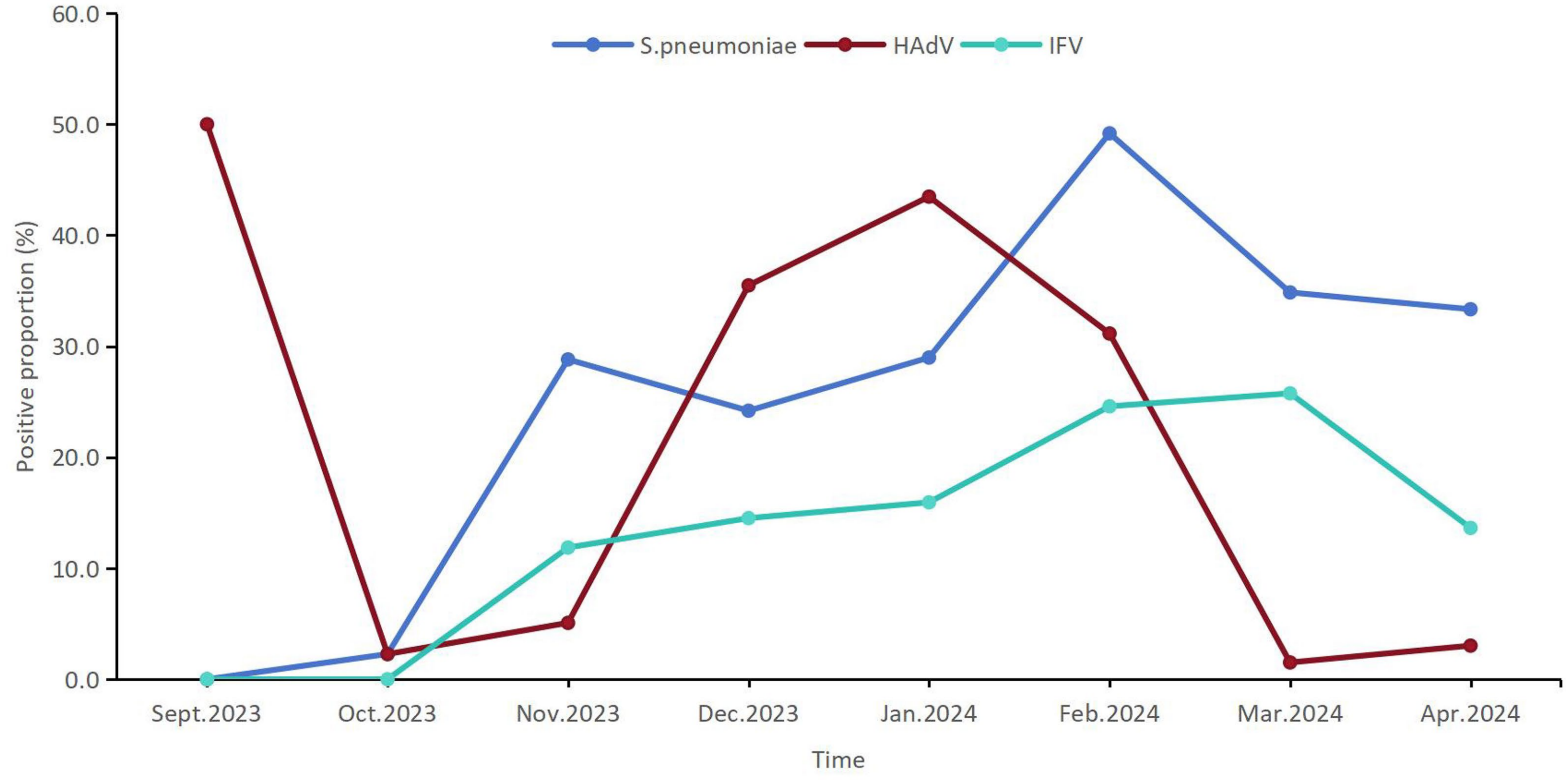
The temporal distribution of the positive proportions of the first three respiratory pathogens detected among children from September 2023 to April 2024 in Shenzhen.

A total of 83 (16.7%) patients were infected with HAdV among 498 pediatric patients with ARIs, which ranked second among the 22 respiratory pathogens. The detection proportion of HAdV infection was relatively high in Jan.2024 (43.5%, 30/69) and Feb.2024 (35.5%, 22/62) (Figure 2).

There were 22 HAdV positive strains from pediatric patients were successfully amplified, including 21 cases of HAdV-3 and 1 case of HAdV-21 base on the phylogenetic analysis of three target genes. The HAdV-21 infected patient was a 9-year-old boy who developed symptoms on May 20, 2024. He presented with fever (peak temperature: 39.2 °C) and nasal discharge but showed no signs of complications such as tracheitis or pneumonia. *S. pneumoniae* co-infection was detected, and the case was classified as mild and managed in the outpatient department.

### Whole genome feature of HAdV-21 strain

**Figure 3:**
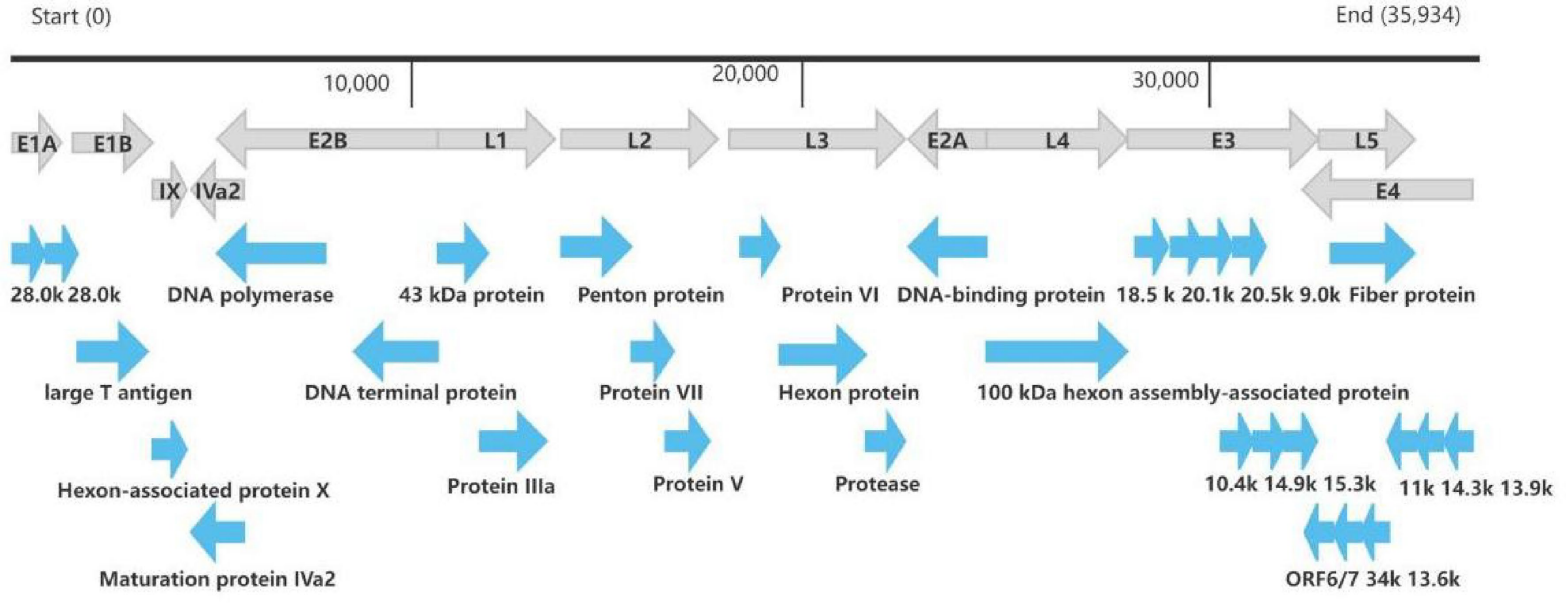
Transcriptional map and genome organization of HAdV-21 strain Shenzhen-2024-5-ILI-1109. The genome was indicated by the black horizontal line marked at 10,000 bp intervals. The transcription units were designated by gray arrows, while blue arrows designated coding regions. Arrows reflected the transcriptional orientation of the coding transcripts.

**Figure 4:**
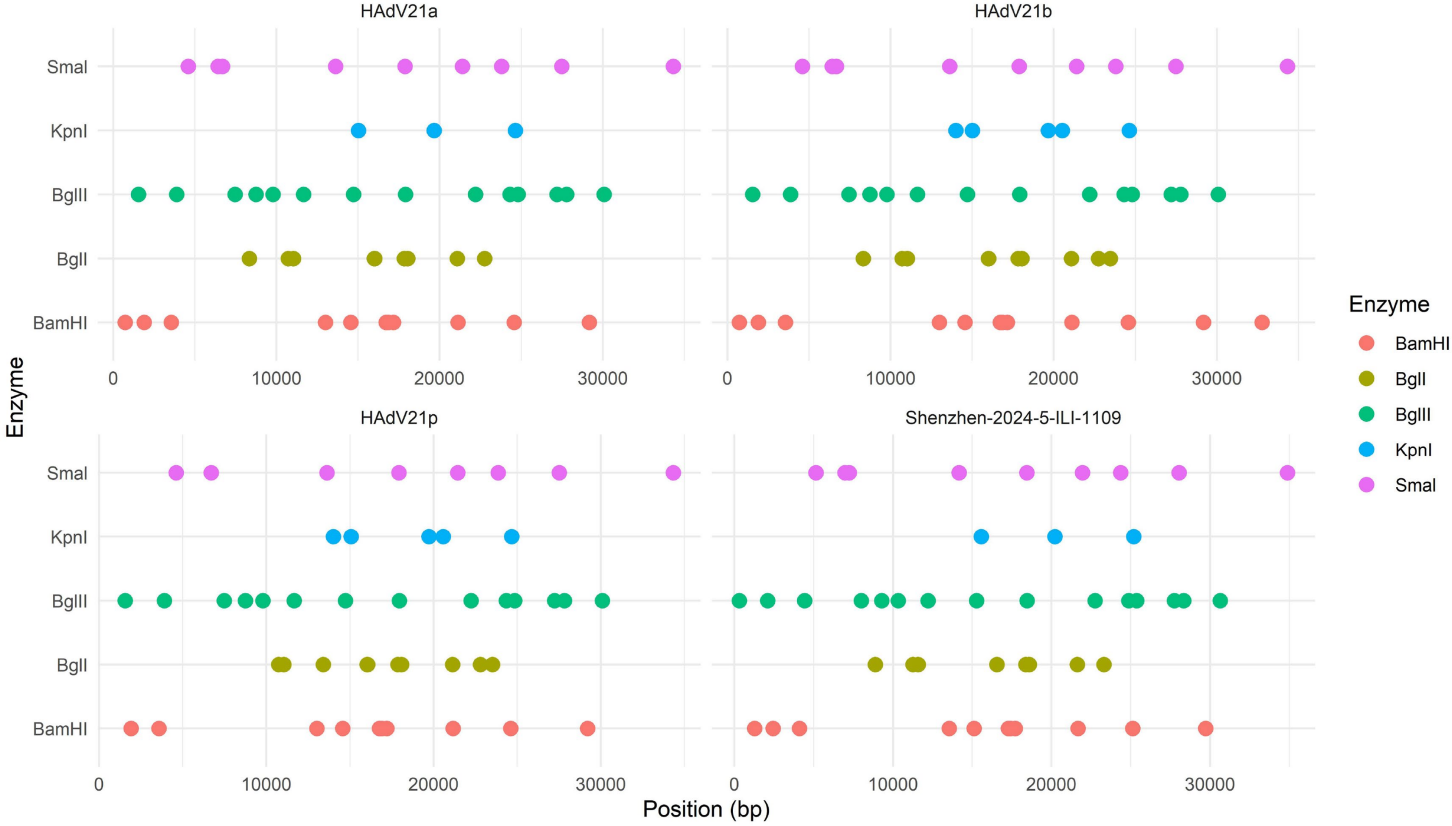
Restriction enzyme analysis of the HAdV-21 strain Shenzhen-2024-5-ILI-1109 sequence using the enzymes BamHI, BglI, BglII, KpnI, and SmaI. Comparison was made with the HAdV-21 strains HAdV-21a NHRC-5, HAdV-21b OHT-006, and HAdV-21p AV-1645.

The whole genome of HAdV-21 strain Shenzhen-2024-5-ILI-1109 was sequenced, annotated, and uploaded to the GenBank database with accession number PX101489. The identified genome was 35,934 bp in length. Figure 3 presented the genomic organization and transcription map for strain Shenzhen-2024-5-ILI-1109 which was similar with that of previously reported HAdV-21 strain GZ09107 in China. The genome of Shenzhen-2024-5-ILI-1109 was composed of 25.4% A, 23.7% T, 25.4% G, and 25.5% C, with a GC content of 50.9% similar to that of HAdV-21 strain GZ09107 (51.2%) (Liu et al., 2022). The strain Shenzhen-2024-5-ILI-1109 was identified as HAdV-21a based on REA of viral genome (Figure 4).

### Phylogenetic analysis of HAdV-21 strain

**Figure 5:**
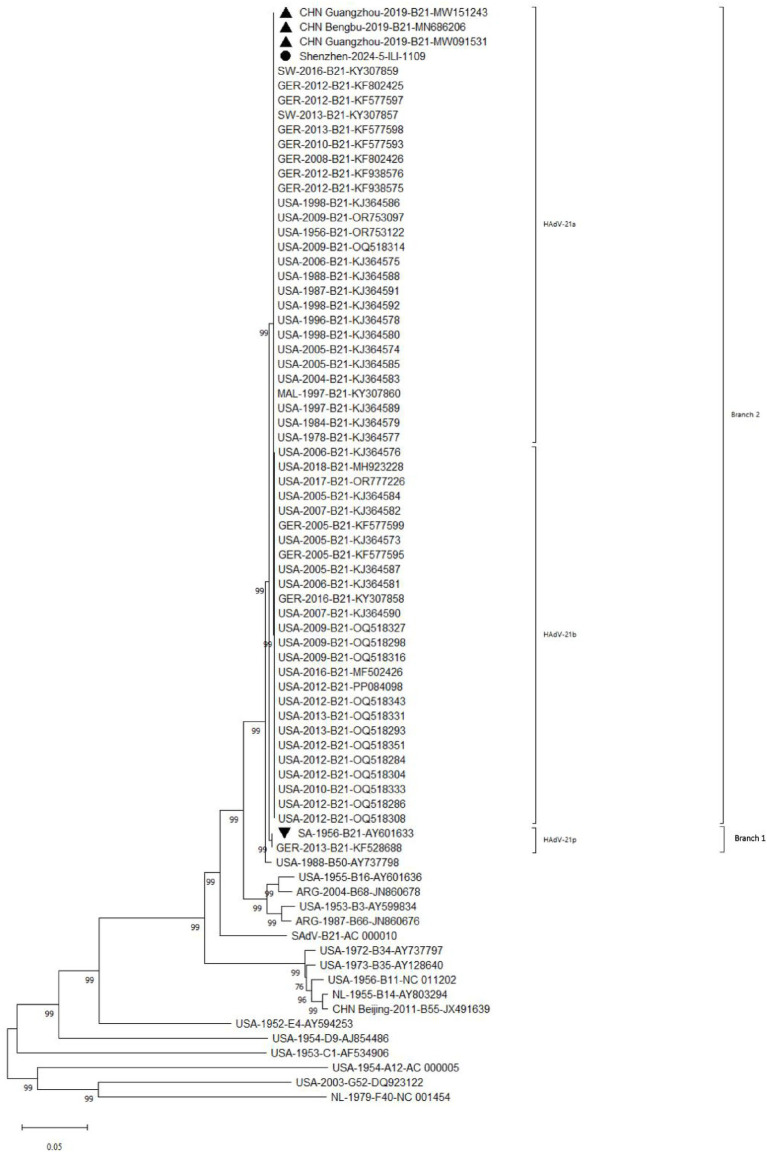
Phylogenetic analysis of HAdV-21 strain Shenzhen-2024-5-ILI-1109. For reference, taxon names included the country of isolation, year of isolation, genome type, and corresponding GenBank accession number. The HAdV-21 strain in this study was marked with “●,” “▲” represented strains isolated from China; “▼” represented reference standard of HAdV-21 isolated in Saudi Arabia in 1956.

Phylogenetic analysis of whole genome showed the HAdV-21 strain Shenzhen-2024-5-ILI-1109 in this study was tightly clustered in one lineage with strains of subtype HAdV-21a (Figure 5). The only two HAdV-21 strains of subtype HAdV-21p, the prototype AV-1645 strain and the GER strain, were clustered in one branch (Branch 1). All other HAdV-21 strains were clustered in Branch 2, which could be subdivided into two lineages, subtypes HAdV-21a and HAdV-21b. The similarity of nucleotides and amino acids of whole genomes was 99.93–99.99% and 99.86–99.97% within a branch, and 99.27 and 98.49% between two branches. The phylogenetic trees based on the three structural proteins including Hexon, Penton base and Fiber also revealed the HAdV-21 strain clustered with strains from subtype HAdV-21a (Supplementary Figure 1). The phylogenetic relationship between HAdV-50 and HAdV-21 was closer than with other types (Figure 5). HAdV-50 was clustered with HAdV-21 strains in the phylogenetic trees based on whole genome, Penton base, and Fiber except Hexon (Figure 5; Supplementary Figure 1).

The amino acids similarity of the whole genomes between the HAdV-21 strain Shenzhen-2024-5-ILI-1109 and other HAdV-21 strains of subtype 21a, 21b, and 21p was 99.85–100.00%, 99.80–99.84%, and 98.63–98.66%, respectively. The nucleotides and amino acids similarity of whole genome of four strains GZ06109, GZ09107, BB/201903, and Shenzhen-2024-5-ILI-1109 from subtype 21a was 100%. The nucleotides and amino acids similarity of Hexon and Fiber gene of Shenzhen-2024-5-ILI-1109 and OHT-006 from subtype 21b was 100%. The amino acids similarity of Penton base and Fiber gene of AV-1645 from subtype 21p and Shenzhen-2024-5-ILI-1109 was 100% (Table 1).

**Table 1:** The nucleotides and amino acids similarity between Shenzhen-2024-5-ILI-1109 and other HAdV-21 strains.

Strain in our study	Other strains	Similarity	Whole genome	Penton base	Hexon	Fiber
Shenzhen-2024-5-ILI-1109	Other strains of subtype 21a	Nucleotide	98.80–100.00%	–	–	–
		Amino acid	99.85–100.00%	–	–	–
	Other strains of subtype 21a in China	Nucleotide	100%	–	–	–
		Amino acid	100%	–	–	–
	Other strains of subtype 21a in USA	Nucleotide	99.90–99.99%	–	–	–
		Amino acid	98.63–99.97%	–	–	–
	Other strains of subtype 21a in Germany	Nucleotide	99.36–99.99%	–	–	–
		Amino acid	98.16–99.97%	–	–	–
	Other strains of subtype 21a in Saudi Arabia	Nucleotide	99.34%	–	–	–
		Amino acid	98.66%	–	–	–
	Other strains of subtype 21a in Malaysia	Nucleotide	99.96%	–	–	–
		Amino acid	99.99%	–	–	–
	Other strains of subtype 21a in Switzerland	Nucleotide	99.97–99.99%	–	–	–
		Amino acid	99.92–99.97%	–	–	–
	Other strains of subtype 21b	Nucleotide	99.90–99.92%	–	–	–
		Amino acid	99.80–99.84%	–	–	–
	Other strains of subtype 21p	Nucleotide	99.34–99.36%	–	–	–
		Amino acid	98.63–98.66%	–	–	–
	CHN_Guangzhou-2019-B21-MW151243 of subtype 21a	Nucleotide	100.00%	100.00%	100.00%	100.00%
		Amino acid	100.00%	100.00%	100.00%	100.00%
	CHN_Guangzhou-2019-B21-MW091531 of subtype 21a	Nucleotide	100.00%	100.00%	100.00%	100.00%
		Amino acid	100.00%	100.00%	100.00%	100.00%
	CHN_Bengbu-2019-B21-MN686206 of subtype 21a	Nucleotide	100.00%	100.00%	100.00%	100.00%
		Amino acid	100.00%	100.00%	100.00%	100.00%
	USA-2016-B21-MF502426 of subtype 21b	Nucleotide	99.92%	99.83%	100.00%	100.00%
		Amino acid	99.84%	99.48%	100.00%	100.00%
	SA-1956-B21-AY601633 of subtype 21p	Nucleotide	99.34%	99.65%	99.50%	99.51%
		Amino acid	98.63%	100.00%	98.88%	100.00%

### Mutation analysis of amino acids between Shenzhen-2024-5-ILI-1109 and other HAdV-21 strains

The whole genome mutation of nucleotides and amino acids of Shenzhen-2024-5-ILI-1109, GZ06109, GZ09107, and BB/201903 with the reference HAdV-21a strain NHRC 5 was compared (Table 2). Most mutations happened in the non-coding regions. The non-synonymous mutations happened in pIX of E1B gene, hypothetical 12.6 kDa protein of E2B gene, Penton base of L2 gene, E2A of E2A gene, 100 kDa protein of L4 gene, 20 kDa protein of E3 gene, or agnoprotein of E4 gene. Compared with GZ09107, GZ06109 and BB/201903, Shenzhen-2024-5-ILI-1109 contained one amino acid insertion mutation in the penton base (GTT, Valine). Compared with Shenzhen-2024-5-ILI-1109, GZ06109 contained one amino acid non-synonymous mutation in large T antigen (H → Y).

**Table 2:** Comparative genomic analysis of HAdV-21a strain Shenzhen-2024-5-ILI-1109 and other strains with the reference HAdV-21a strain NHRC 5.

Region	Gene	Position	Mutation in DNA				Mutation in AA			
			CHN Guangzhou-2019-B21-MW151243	CHN Guangzhou-2019-B21-MW091531	CHN Bengbu-2019-B21-MN686206	Shenzhen-2024-5-ILI-1109	CHN Guangzhou-2019-B21-MW151243	CHN Guangzhou-2019-B21-MW091531	CHN Bengbu-2019-B21-MN686206	Shenzhen-2024-5-ILI-1109
5’-UTR	NCR	164	C → G	C → G	C → G	C → G	**L → V**	**L → V**	**L → V**	**L → V**
	NCR	256	▼T	▼T	▼T	▼T	▼S	▼S	▼S	▼S
	NCR	266	C → T	C → T	C → T	C → T	**A → V**	**A → V**	**A → V**	**A → V**
	NCR	328	-	-	-	C → T	-	-	-	**R → C**
E1B	Small T antigen	1950	C → T	-	-	-	ds	-	-	**-**
	Large T antigen	1950	C → T	-	-	-	H → Y	-	-	**-**
	pIX	3,785	G → C	G → C	G → C	G → C	**C → S**	**C → S**	**C → S**	**C → S**
E2B	Hypothetical 12.6 kDa protein	8,271	G → T	G → T	G → T	G → T	**R → S**	**R → S**	**R → S**	**R → S**
L1	NCR	10,577	T → C	T → C	T → C	T → C	**F → S**	**F → S**	**F → S**	**F → S**
	NCR	10,578	C → G	C → G	C → G	C → G	**F → S**	**F → S**	**F → S**	**F → S**
	NCR	10,579	▲G	▲G	▲G	G → T	▲V	▲V	▲V	**V → F**
	NCR	10,838	G → T	G → T	G → T	G → T	ds	ds	ds	ds
L2	Penton base	13,980	▼TGTT	▼TGTT	▼GT	▼GTT	▼FV	▼FV	ds	▼V
	NCR	15,542	▼AAAA	▼AAAA	▼AAAAAAAAAA	▼AAA	▼K	▼K	▼KKK	▼K
E2A	E2A	23,079	A → G	A → G	A → G	A → G	**K → R**	**K → R**	**K → R**	**K → R**
L4	100 kDa protein	25,040	A → C	A → C	A → C	A → C	**I → L**	**I → L**	**I → L**	**I → L**
E3	20 kDa protein	28,764	G → A	-	-	-	**G → D**	-	-	-
	NCR	29,207	▼T	-	-	-	▼N	-	-	-
	NCR	29,788	▼T	▼T	▼T	▼T	▼N	▼N	▼N	▼N
	NCR	30,092	▼TT	▼TT	▼TT	▼TT	▼SF	▼SF	▼SF	▼SF
	NCR	31,216	▼A	▼A	▼A	▼A	▼I	▼I	▼I	▼I
L5	NCR	32,374	▼A	▼A	▼A	▼A	▼K	▼K	▼K	▼K
E4	Agnoprotein	34,062	G → T	G → T	G → T	G → T	**R → I**	**R → I**	**R → I**	**R → I**
3’-UTR	NCR	35,116	▼AA	▼AA	▼AA	▼AA	▼K	▼K	▼K	▼K

NCR, non-coding region; ▼, insertion; ▲, deletion; −, no change or not applicable; dS, synonymous; AA, amino acid. The bold values showed amino acids of non-synonymous mutation.

Hexon and Fiber gene were the two major neutralization antigens of HAdV. The Fiber proteins were highly conserved in all HAdV-21 strains with a variation of only one or two amino acids (Supplementary Figure 2). Detailed phylogenetic analysis for Hexon also indicated the existence of the two branches (Supplementary Figure 1). Branch 1 included strains of subtype 21p, and branch 2 included strains of subtype 21a and 21b. Multiple sequence alignments revealed changes between the two branches in HVR3, HVR4, and HVR7 except HVR1, HVR2, HVR5, and HVR6. Among strains of the same branch, there was no amino acid substitution. Strikingly, compared with HAdV-21a and HAdV-21p, HAdV-21b contained a 16 amino acid peptide insertion mutation, _313_TEAAKAAAIAKANIVV_328_, and a 3 amino acid deletion mutation, _361_AET_363_, in the Penton base. Multiple alignments also revealed the mutations between the two branches in Fiber protein.

## Discussion

There were 73.5% patients infected with one or more of the 22 pathogens among pediatric patients with ARIs from September 2023 to April 2024 in Shenzhen, and the most frequently detected pathogens were *S. pneumoniae*, HAdV, and IFV. The HAdV-21 strain was classified as subtype 21a with genomes closely related to other strains found in China. Phylogenetic analysis for whole genome and major antigen proteins showed that global HAdV-21 strains could be classified into two branches, branch 1 including genotype 21p, branch 2 including genotype 21a and 21b. There was a single amino acid insertion (GTT, Valine) in the Penton base protein of the Shenzhen-2024-5-ILI-1109 strain, compared to previously circulating HAdV-21 strains in China. There were three highly variable regions (HVR3, HVR4, and HVR7) in the Hexon protein that varied between two branches.

The high pathogen positive proportion among children with ARIs from September 2023 to April 2024 in Shenzhen was consistent with the findings of previous studies in multiple cities of China (Li et al., 2021; Zhao et al., 2024; Zhu et al., 2025), underscoring the importance of continuous surveillance for multiple respiratory pathogens. The high detection rate of *S. pneumoniae* among children was close to that reported in studied from China (38.5%) (Li et al., 2021), may be related to the high carriage rate in children and secondary infections. Some studies have indicated that *S. pneumoniae* often co-infected with viruses and exacerbated disease severity (Palacios et al., 2009), which aligned with the high co-infection rate observed in this study. The high prevalence of IFV highlighted the importance of annual influenza vaccination in the pediatric population, which has clear public health significance for reducing the societal healthcare burden and the incidence of severe complications. It is noteworthy that this study also found a high rate of mixed pathogen infections. Research has shown that viral-bacterial co-infections can have synergistic pathogenic effects (Niu et al., 2025), for example, IFV damaging the respiratory epithelial barrier can easily lead to secondary *S. pneumoniae* infections (Kash and Taubenberger, 2015). Therefore, the interpretation of etiological diagnostic results should be combined with clinical manifestations and imaging findings in clinical practice, especially for severely ill children, where the possibility of mixed infection should be considered and targeted treatment provided.

This study showed that the positive proportion of HAdV in children patients in Shenzhen from 2023 to 2024 was relatively high, which was consistent with the results of many studies worldwide (Chen et al., 2022; Fukuda et al., 2024; Wang et al., 2023; Wang et al., 2022). This may be related to the immune debt caused by the COVID-19 pandemic or the change in the dominant genotype of HAdV (Alzaydi et al., 2024; Lee et al., 2024; Perez et al., 2022). A study conducted in Guangzhou similarly found that HAdV was one of the major pathogens among pediatric inpatients with ARIs and was associated with a higher proportion of severe pneumonia and febrile cases (Chen et al., 2016). The results of HAdV typing revealed a predominance of genotype B3 in 2023–2024 in Shenzhen, with only a single strain of HAdV-21 detected. The dominant type of HAdV in Shenzhen was relatively consistent with the results in China (Jin et al., 2025). The low prevalence of HAdV-21 may signify low immunity against this type in the general population, increasing its potential to cause an epidemic.

Phylogenetic analysis for whole genome and major antigen proteins showed that global HAdV-21 strains could be classified into two branches, branch 1 including genotype 21p, branch 2 including genotype 21a and 21b, which was consistent with earlier research results (Liu et al., 2022). Sequencing and annotation of the genome revealed similar structure to other strains of HAdV species B (Tang et al., 2013). The HAdV-21 isolate genome in this study constituted a clade with HAdV-21a, and had high genome identity with HAdV-21a strains BB/201903, GZ09107, and GZ06109. These findings suggested that the prevalent HAdV-21a isolates in China shared a high degree of genetic relatedness and likely originated from a common source, though available data remained insufficient to pinpoint its origin. This molecular epidemiological pattern further indicated that HAdV-21a has emerged as a significant cause of respiratory infections in Chinese children and may possess region-specific adaptive mutations.

The observed single amino acid insertion (GTT, Valine) in the Penton base protein of the Shenzhen-2024-5-ILI-1109 strain, compared to previously circulating HAdV-21 strains in China, may have implications for viral infectivity and tropism. The penton base protein is a key structural protein of the HAdV capsid, playing a central role not only in viral assembly and stability but also serving as a critical molecule mediating virus internalization (Kulanayake and Tikoo, 2021). This protein interacts with host cell surface integrins through its RGD motif, initiating virus endocytosis. Therefore, any variation in this region, particularly an insertion event that may induce conformational changes in the protein, could significantly alter the virus’s cell tropism and infection efficiency. Valine, as a nonpolar, hydrophobic amino acid, may enhance the flexibility or stability of local loop structures through its insertion, potentially increasing the affinity of this protein for host cell receptors and thereby potentially enhancing the virus’s infectivity. Furthermore, this variation may represent an immune escape mechanism. The penton protein is an important target for neutralizing antibodies (Rux and Burnett, 2004). An amino acid insertion near a key antigenic site could alter surface epitopes, preventing neutralizing antibodies generated from prior infections from effectively recognizing and neutralizing this variant strain. This provides the possibility for the virus to establish a transmission advantage among populations with some level of pre-existing immunity. Future studies urgently need to functionally validate the specific impacts of this mutation on viral receptor affinity, cell tropism, replication capacity, and immune escape through reverse genetics, pseudovirus neutralization assays, and cell infection models.

Most serotype-specific neutralizing epitopes of HAdV were located in multiple HVRs within the hexon protein (Kulanayake and Tikoo, 2021), and there were three highly variable regions (HVR3, HVR4, and HVR7) in the hexon protein that varied between two branches, possibly contributing to the drift of neutralization profiles. Strains of subtypes HAdV-21a and 21b of branch 2 have predominated since the 1960s. It is important to note that the neutralization epitopes of branch 2 strains remain virtually unchanged. We believe our findings and the isolated HAdV-21a strain in Shenzhen could be helpful in novel HAdV-21 vaccine and drug development. Amino acid variations in these regions may directly affect the viral antigenicity, leading to immune escape. The branch-specific mutation patterns observed in this study suggested that antibody responses may vary significantly across different branches, which has important implications for vaccine development.

There were still some limitations in this study. Firstly, the study detected only one strain of HAdV-21, which was isolated from an outpatient setting with no severe cases included, may lead to deviations in the understanding of HAdV-21 infection, especially the epidemiological characteristics. Secondly, as the HAdV samples were not isolated and cultured, the viral load was relatively low, resulting in fewer successfully amplified HAdV gene sequences, and virus isolation and culture experiments will continue to be carried out to discover more HAdV-21 strains in the future. Thirdly, this study is merely a preliminary analysis of the HAdV-21 gene sequence, and it can be combined with structural biology or protein experiments to further analyze the possible impact of amino acid mutations on protein structure or function.

## Conclusion

The most frequently detected pathogens among pediatric patients with ARIs from September 2023 to April 2024 in Shenzhen were *S. pneumoniae*, HAdV, and IFV. HAdV-21 infection of children was firstly reported in Shenzhen, China. The global HAdV-21 strains could be classified into two branches, and the HAdV-21 strain in this study was classified as subtype 21a with genomes closely related to other strains found in China. Mutations in three highly variable regions between the two branches may alter antigenic profiles. Our findings have increased the database of HAdV-21 gene sequences and are of great significance for understanding its epidemiological characteristics and the development of related vaccines.

## Data Availability

The datasets presented in this study can be found in online repositories. The names of the repository/repositories and accession number(s) can be found in the article/Supplementary material.

## Footnotes

^1^ https://mafft.cbrc.jp/alignment/software/

^2^ http://www.softpedia.com/get/Science-CAD/BioEdit.shtml
