# Supplementary material for: First identification of human adenovirus subtype 21a in Shenzhen, China with high-throughput sequencing
Source: Front Microbiol. 2025 Oct 13;16:1692162. doi: 10.3389/fmicb.2025.1692162 (PMC12554774; doi:10.3389/fmicb.2025.1692162)
Supplement: Supplementary file 1 [file Data_Sheet_1.docx]

**Supplemental Material**

**Supplementary Table 1. Universal primers for the detection, typing, and sequencing of HAdV**

| Gene | Position | Lengthª (bp) | Primer | Primer sequence | Position^a^ | PCR product | PCR condition |
| --- | --- | --- | --- | --- | --- | --- | --- |
| Penton base | 13,904-15,538 | 1,635 | Penton-F | 5'-CTATCAGAACGACCACAGCAACTT-3’ | 14,152-14,175 | 1,253 bp | 34 cycles of 94°C for 30s; 52°C for 30s; and 72°C for 100s |
|  |  |  | Penton-R | 5'-TCCCGTGATCTGTGAGAGCRG-3’ | 15,384-15,404 |  |  |
| Hexon | 18,422-21,256 | 2,835 | HVR-F | 5'-CAGGATGCTTCGGAGTACCTGAG-3' | 18,473-18,495 | 1,685 bp |  |
|  |  |  | HVR-R | 5'-TTTCTGAAGTTCCACTCGTAGGTGTA-3’ | 20,132-20,157 |  |  |
| Fiber | 31,301-32,260 | 960(1278b) | Fiber-F | 5'-CCCTCTTCCCAACTCTGGTA-3’ | 31,180-31,199 | 1,153bp |  |
|  |  |  | Fiber-R | 5’-GGGGAGGCAAAATAACTACTCG-3' | 32,311-32,332 | (1,519 bpb) |  |
|  |  | 1746c | Fiber-CR | 5’-GAGGTGGCAGGTTGAATACTAG-3' | 32.311-32.332 | 2027 bpc |  |

Sequences, genome locations, and the resultant predicted PCR product sizes were noted for each of the three major capsid protein genes. Fiber-CR was the species C-specific optimal primer due to their divergence from other HAdVs.

^a^Positions were in the reference genome of HAdV-B3 (GenBank acc. no. DQ099432).

^b^The length of HAdV-4 fiber gene, for reference.

^c^The length of the PCR product of HAdV-C fiber gene, for reference.

**Supplementary Table 2. Sequences of human adenovirus species A–G used for phylogenetic analysis**

| No. | Strain Name in GenBank | Country/Province | Regional abbreviation | Collection Date | Genotype | Accession Number | | |
| --- | --- | --- | --- | --- | --- | --- | --- | --- |
|  |  |  |  |  |  | P | H | F |
| 1 | OHT-006 | the United States | USA | 2016.4 | HAdV-B21 | MF502426 | MF502426 | MF502426 |
| 2 | HAdV-B21/USA/9J5/2012 | the United States | USA | 2012.4 | HAdV-B21 | PP084098 | PP084098 | PP084098 |
| 3 | HAdV-B21/USA/7K4/1956 | the United States | USA | 1956.2 | HAdV-B21 | OR753122 | OR753122 | OR753122 |
| 4 | HAdV-B21/USA/5L10/2009 | the United States | USA | 2009.12 | HAdV-B21 | OR753097 | OR753097 | OR753097 |
| 5 | HAdV-B21/USA/10E6/2012 | the United States | USA | 2012.12 | HAdV-B21 | OQ518351 | OQ518351 | OQ518351 |
| 6 | HAdV-B21/USA/9I6/2012 | the United States | USA | 2012.4 | HAdV-B21 | OQ518343 | OQ518343 | OQ518343 |
| 7 | HAdV-B21/USA/6K2/2010 | the United States | USA | 2010.6 | HAdV-B21 | OQ518333 | OQ518333 | OQ518333 |
| 8 | HAdV-B21/USA/10L3/2013 | the United States | USA | 2013.4 | HAdV-B21 | OQ518331 | OQ518331 | OQ518331 |
| 9 | HAdV-B21/USA/2B2/2009 | the United States | USA | 2009.4 | HAdV-B21 | OQ518327 | OQ518327 | OQ518327 |
| 10 | HAdV-B21/USA/2B10/2009 | the United States | USA | 2009.5 | HAdV-B21 | OQ518316 | OQ518316 | OQ518316 |
| 11 | HAdV-B21/USA/4B5/2009 | the United States | USA | 2009.9 | HAdV-B21 | OQ518314 | OQ518314 | OQ518314 |
| 12 | HAdV-B21/USA/9M3/2012 | the United States | USA | 2012.6 | HAdV-B21 | OQ518308 | OQ518308 | OQ518308 |
| 13 | HAdV-B21/USA/10E4/2012 | the United States | USA | 2012.12 | HAdV-B21 | OQ518304 | OQ518304 | OQ518304 |
| 14 | HAdV-B21/USA/4C7/2009 | the United States | USA | 2009.9 | HAdV-B21 | OQ518298 | OQ518298 | OQ518298 |
| 15 | HAdV-B21/USA/10L4/2013 | the United States | USA | 2013.4 | HAdV-B21 | OQ518293 | OQ518293 | OQ518293 |
| 16 | HAdV-B21/USA/9M4/2012 | the United States | USA | 2012.6 | HAdV-B21 | OQ518286 | OQ518286 | OQ518286 |
| 17 | HAdV-B21/USA/10E5/2012 | the United States | USA | 2012.12 | HAdV-B21 | OQ518284 | OQ518284 | OQ518284 |
| 18 | HAdV-B21/USA/14A2/2017 | the United States | USA | 2017.9 | HAdV-B21 | OR777226 | OR777226 | OR777226 |
| 19 | HAdVB/USA_NY/10208/2018/P21H21F21 | the United States | USA | 2018 | HAdV-B21 | MH923228 | MH923228 | MH923228 |
| 20 | GZ09107.1 | China Guangzhou | CHN_Guangzhou | 2019.9 | HAdV-B21 | MW151243 | MW151243 | MW151243 |
| 21 | GZ06109.1 | China Guangzhou | CHN_Guangzhou | 2019.6 | HAdV-B21 | MW091531 | MW091531 | MW091531 |
| 22 | VRDL T98-1269 | the United States | USA | 1998 | HAdV-B21 | KJ364592 | KJ364592 | KJ364592 |
| 23 | VRDL T87-0342 | the United States | USA | 1987 | HAdV-B21 | KJ364591 | KJ364591 | KJ364591 |
| 24 | NHRC 91447 | the United States | USA | 2007 | HAdV-B21 | KJ364590 | KJ364590 | KJ364590 |
| 25 | VRDL T97-1745 | the United States | USA | 1997 | HAdV-B21 | KJ364589 | KJ364589 | KJ364589 |
| 26 | CDC V2148A | the United States | USA | 1988 | HAdV-B21 | KJ364588 | KJ364588 | KJ364588 |
| 27 | PEL0066 | the United States | USA | 2005 | HAdV-B21 | KJ364587 | KJ364587 | KJ364587 |
| 28 | NHRC 10030 | the United States | USA | 1998 | HAdV-B21 | KJ364586 | KJ364586 | KJ364586 |
| 29 | NHRC 71252 | the United States | USA | 2005 | HAdV-B21 | KJ364585 | KJ364585 | KJ364585 |
| 30 | NHRC 71227 | the United States | USA | 2005 | HAdV-B21 | KJ364584 | KJ364584 | KJ364584 |
| 31 | NHRC 71139 | the United States | USA | 2004 | HAdV-B21 | KJ364583 | KJ364583 | KJ364583 |
| 32 | NHRC 64589 | the United States | USA | 2007 | HAdV-B21 | KJ364582 | KJ364582 | KJ364582 |
| 33 | NHRC 52331 | the United States | USA | 2006 | HAdV-B21 | KJ364581 | KJ364581 | KJ364581 |
| 34 | NHRC 20007 | the United States | USA | 1998 | HAdV-B21 | KJ364580 | KJ364580 | KJ364580 |
| 35 | CDC V1375E | the United States | USA | 1984 | HAdV-B21 | KJ364579 | KJ364579 | KJ364579 |
| 36 | NHRC 5 | the United States | USA | 1996 | HAdV-B21 | KJ364578 | KJ364578 | KJ364578 |
| 37 | CDC RU8176 | the United States | USA | 1978 | HAdV-B21 | KJ364577 | KJ364577 | KJ364577 |
| 38 | NHRC 44288 | the United States | USA | 2006 | HAdV-B21 | KJ364576 | KJ364576 | KJ364576 |
| 39 | NHRC 63218 | the United States | USA | 2006 | HAdV-B21 | KJ364575 | KJ364575 | KJ364575 |
| 40 | NHRC 32493 | the United States | USA | 2005 | HAdV-B21 | KJ364574 | KJ364574 | KJ364574 |
| 41 | NHRC 32389 | the United States | USA | 2005 | HAdV-B21 | KJ364573 | KJ364573 | KJ364573 |
| 42 | Human adenovirus 21 | Germany | GER | 2013 | HAdV-B21 | KF528688 | KF528688 | KF528688 |
| 43 | NCH248 | the United States | USA | 2012 | HAdV-B21 | - | - | KM654553 |
| 44 | NHRC 2670 | the United States | USA | 2014 | HAdV-B21 | - | - | KM654552 |
| 45 | NHRC 26759 | the United States | USA | 2014 | HAdV-B21 | - | - | KM654551 |
| 46 | NHRC 66008 | the United States | USA | 2009 | HAdV-B21 | - | - | KM654550 |
| 47 | NHRC 64983 | the United States | USA | 2008 | HAdV-B21 | - | - | KM654549 |
| 48 | NHRC 72539 | the United States | USA | 2008 | HAdV-B21 | - | - | KM654548 |
| 49 | NHRC 72736 | the United States | USA | 2009 | HAdV-B21 | - | - | KM654547 |
| 50 | NHRC 23858 | the United States | USA | 2008 | HAdV-B21 | - | - | KM654546 |
| 51 | NHRC 91569 | the United States | USA | 2008 | HAdV-B21 | - | - | KM654545 |
| 52 | NHRC 74308 | the United States | USA | 2011 | HAdV-B21 | - | - | KM654544 |
| 53 | NHRC 35931 | the United States | USA | 2010 | HAdV-B21 | - | - | KM654543 |
| 54 | human/CHN/BB/201903/21 | China Bengbu | CHN_Bengbu | 2019.3 | HAdV-B21 | MN686206 | MN686206 | MN686206 |
| 55 | Sibu-97 | Malaysia | MAL | 1997 | HAdV-B21 | KY307860 | KY307860 | KY307860 |
| 56 | LRTI-9 | Switzerland | SW | 2016 | HAdV-B21 | KY307859 | KY307859 | KY307859 |
| 57 | LRTI-8 | Germany | GER | 2016 | HAdV-B21 | KY307858 | KY307858 | KY307858 |
| 58 | LRTI-7 | Switzerland | SW | 2013 | HAdV-B21 | KY307857 | KY307857 | KY307857 |
| 59 | LRTI-1 | Germany | GER | 2005 | HAdV-B21 | KF577599 | KF577599 | KF577599 |
| 60 | LRTI-4 | Germany | GER | 2012 | HAdV-B21 | KF938576 | KF938576 | KF938576 |
| 61 | LRTI-4 | Germany | GER | 2012 | HAdV-B21 | KF938575 | KF938575 | KF938575 |
| 62 | LRTI-2 | Germany | GER | 2008 | HAdV-B21 | KF802426 | KF802426 | KF802426 |
| 63 | LRTI-5 | Germany | GER | 2012 | HAdV-B21 | KF802425 | KF802425 | KF802425 |
| 64 | LRTI-6 | Germany | GER | 2013 | HAdV-B21 | KF577598 | KF577598 | KF577598 |
| 65 | LRTI-5 | Germany | GER | 2012 | HAdV-B21 | KF577597 | KF577597 | KF577597 |
| 66 | LRTI-1 | Germany | GER | 2005 | HAdV-B21 | KF577595 | KF577595 | KF577595 |
| 67 | LRTI-3 | Germany | GER | 2010 | HAdV-B21 | KF577593 | KF577593 | KF577593 |
| 68 | AV-1645 | the United States | USA | 2000 | HAdV-B21 | - | AY008279 | - |
| 69 | AV-1645 | Saudi Arabia | SA | 1956 | HAdV-B21 | AY601633 | AY601633 | AY601633 |
| 70 | Wan; ATCC VR-1502 | the United States | USA | 1988 | HAdV-B50 | AY737798 | AY737798 | AY737798 |
| 71 | GB（prototype) | the United States | USA | 1953 | HAdV-B3 | AY599834 | AY599834 | AY599834 |
| 72 | 87-922 | Argentina | ARG | 1987 | HAdV-B66 | JN860676 | JN860676 | JN860676 |
| 73 | ch. 79 | the United States | USA | 1955 | HAdV-B16 | AY601636 | AY601636 | AY601636 |
| 74 | Arg 827/04 | Argentina | ARG | 2004 | HAdV-B68 | JN860678 | JN860678 | JN860678 |
| 75 |  |  |  |  | SAdV-B21 | AC_000010 | AC_000010 | AC_000010 |
| 76 | Compton | the United States | USA | 1972 | HAdV-B34 | AY7377971 | AY7377971 | AY7377971 |
| 77 | Holden | the United States | USA | 1973 | HAdV-B35 | AY128640 | AY128640 | AY128640 |
| 78 | Slobitski | the United States | USA | 1956 | HAdV-B11 | NC_011202 | NC_011202 | NC_011202 |
| 79 | de Wit | Netherlands | NL | 1955 | HAdV-B14 | AY803294 | AY803294 | AY803294 |
| 80 | BJ01 | China Beijing | CHN_Beijing | 2011 | HAdV-B55 | JX491639 | JX491639 | JX491639 |
| 81 | R1-67 | the United States | USA | 1952 | HAdV-E4 | AY594253 | AY594253 | AY594253 |
| 82 | Hicks | the United States | USA | 1954 | HAdV-D9 | AJ854486 | AJ854486 | AJ854486 |
| 83 | Adenoid 71 | the United States | USA | 1953 | HAdV-C1 | AF534906 | AF534906 | AF534906 |
| 84 | Huie | the United States | USA | 1954 | HAdV-A12 | AC_000005 | AC_000005 | AC_000005 |
| 85 | Dugan | Netherlands | NL | 1979 | HAdV-F40 | NC_001454 | NC_001454 | NC_001454 |
| 86 | T03-2244 | the United States | USA | 2003 | HAdV-G52 | DQ923122 | DQ923122 | DQ923122 |
| 87 | Shenzhen-2024-5-ILI-1109^#^ | China Shenzhen | CHN_Shenzhen | 2024 | HAdV-B21 | PX101489 | PX101489 | PX101489 |

^#^ Isolated in this study.

**
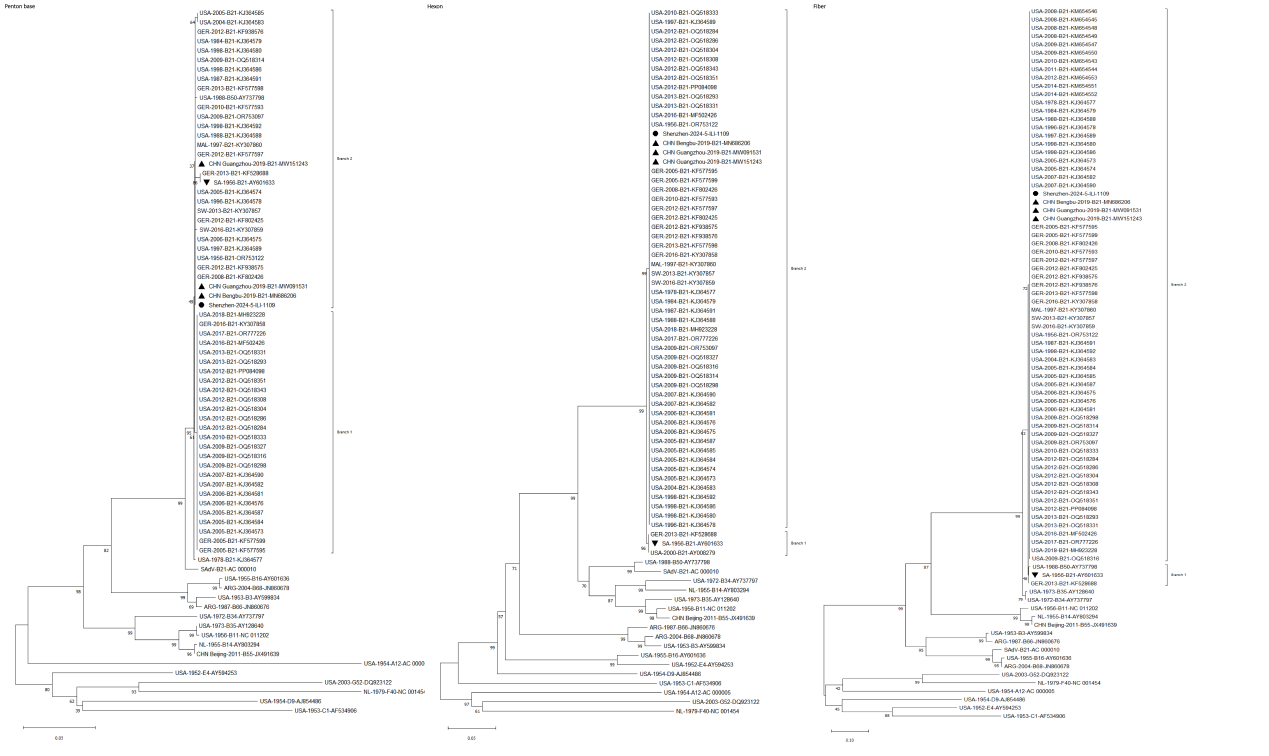
**

**Supplementary Figure 1.** Phylogenetic analysis of HAdV-21 strain Shenzhen-2024-5-ILI-1109. For reference, taxon names include the country of isolation, year of isolation, genome type, and corresponding GenBank accession number. The HAdV-21 strain in this study was marked with “●”, “▲” represented strains isolated from China; “▼” represented reference standard of HAdV-21 isolated in Saudi Arabia in 1956.

**
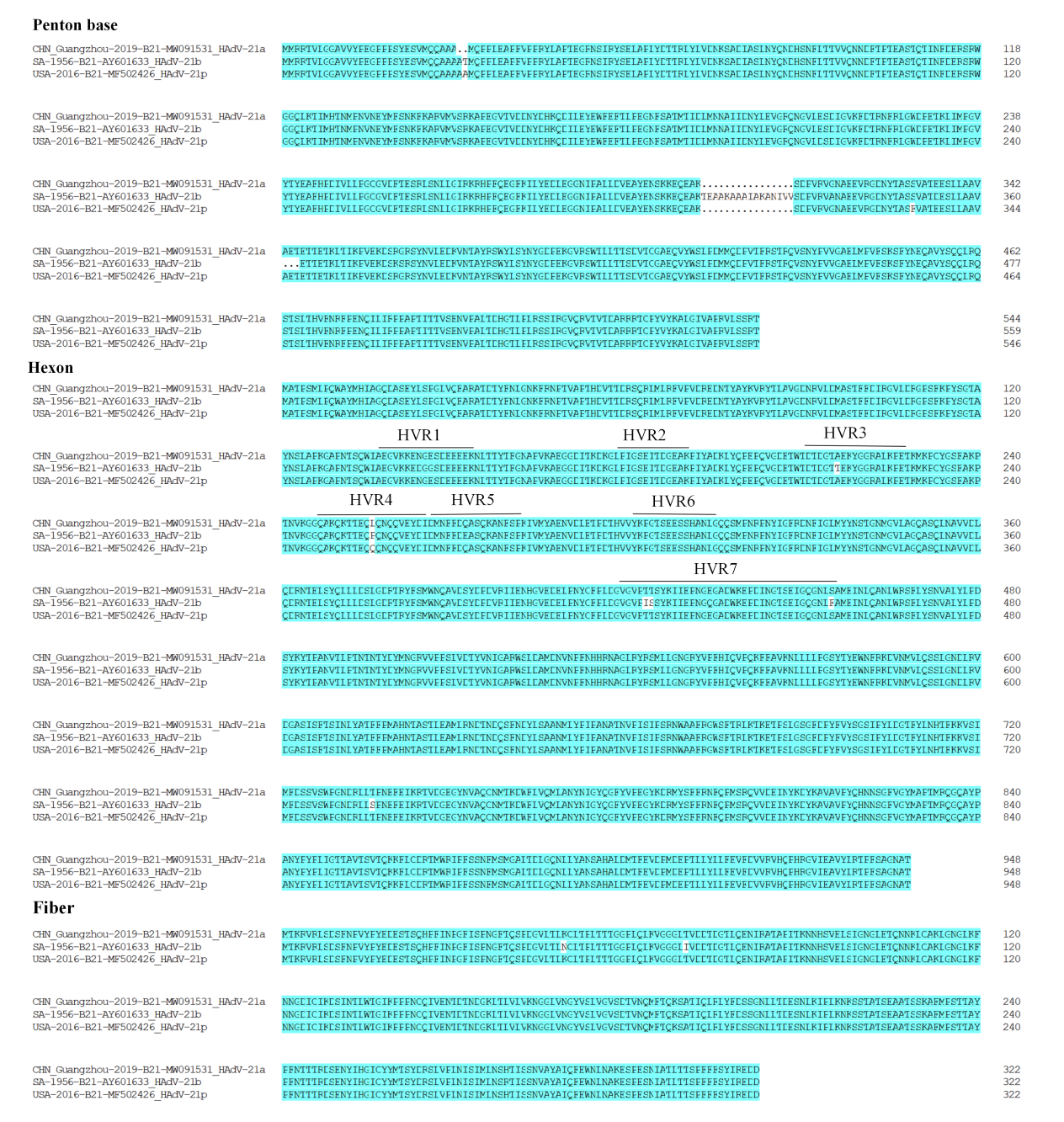
**

**Supplementary Figure 2.** Multiple sequence alignment of HAdV-21 major capsid proteins. Multiple sequence alignment of the full proteins penton base, hexon and fiber from HAdV-21 subtypes 21a (GZ06109), 21b (OHT-006) and 21p (AV-1645). Seven hypervariable regions of hexon were shown. The number showed the position of amino acid in the protein.
